# A novel p.Val244Leu mutation in *MFN2* leads to Charcot-Marie-Tooth disease type 2

**DOI:** 10.1186/s13052-016-0237-8

**Published:** 2016-03-08

**Authors:** Yuan Yang, Ling Li

**Affiliations:** Clinical Class 2, Grade 2012 in Soochow University, Suzhou, 215006 China; Pediatric Neurology in Xinhua Hospital Affiliated to Shanghai Jiaotong University School of Medicine, Shanghai, 200092 China

**Keywords:** Charcot-Marie-Tooth (CMT) disease, MFN2, Missense mutation, Hereditary

## Abstract

**Background:**

Charcot-Marie-Tooth (CMT) disease is one of the most common hereditary peripheral neuropathy. The major clinical features of CMT are progressive muscle weakness of distal extremities and sensory loss. *MFN2* encodes a GTPase dynamin-like protein mitofusin 2 and plays an essential role in mitochondrial functions. In previous studies, *MFN2* mutations have been linked to neurological disorders including CMT type 2 (CMT2). Here, we report a novel mutation in *MFN2* which leads to CMT 2.

**Case presentation:**

We report a 4-year-old Chinese boy with CMT symptoms including foot-drop gait, running difficulties, frequent falls, slowly progressive atrophy of lower legs with a mildly foot deformity. Nerve conduction velocity study (NCVS) found that no compound motor action potential (CMAP) was elicited in the nervi suralis and tibial nerve. Moreover, the sensory nerve action potential (SNAP) of the nervi suralis was not elicited, which means the peripheral nerves of his lower limbs were damaged. Targeted next-generation sequencing identified a novel heterozygous mutation c.730G > C (p.Val244Leu) in *MFN2* in the patient but not in his parents, suggesting that this mutation likely occurred *de novo*. c.730G > C (p.Val244Leu) in *MFN2* is a likely pathogenic mutation for CMT2.

**Conclusion:**

The c.730G > C (p.Val244Leu) mutation in *MFN2* is a likely pathogenic mutation for CMT2.

## Background

Charcot-Marie-Tooth (CMT) disease, also known as hereditary motor sensory neuropathy (HMSN),is one of the most common hereditary peripheral neuropathy with an estimated prevalence of 1 in 2500 [1, 2]. The major clinical features of CMT are progressive muscle weakness of distal extremities and sensory loss [3]. According to the clinical symptoms and electrophysiological characteristics, CMT is categorized into type 1 (CMT1) and type 2 (CMT2) [4]. CMT1 is characterized by hypertrophic demyelination with slow nerve conduction velocities (NCVs) (median nerve conduction velocity <38 m/s), while CMT2 is characterized by normal or mildly reduced NCVs (median nerve conduction velocity >38 m/s) along with axonopathy [1, 4].

*MFN2* is located on the short arm of chromosome 1 and encodes mitofusin 2, a GTPase dynamin-like protein of the outer mitochondrial membrane. It plays an essential role in mitochondrial functions including fusion, axonal transport, interoranellar communication and mitophagy [5, 6]. In previous studies, *MFN2* mutations have been linked to neurological disorders including CMT2 (named as CMT2A2, OMIM#609260). To date, more than 100 *MFN2* mutations have been reported to be associated with CMT2 [6, 7]. Among them, there is just one mutation at the 730^th^ nucleotide in the encoding region (c.730G > A/p.Val244Met) [8].

Here, we reported a 4 year-old-boy with CMT2 symptoms including muscle weakness, difficulty in running and frequent fall. Targeted next-generation sequencing identified a novel heterozygous mutation c.730G > C (p.Val244Leu) in *MFN2*, which occurred *de novo*.

## Case presentation

**Fig. 1:**
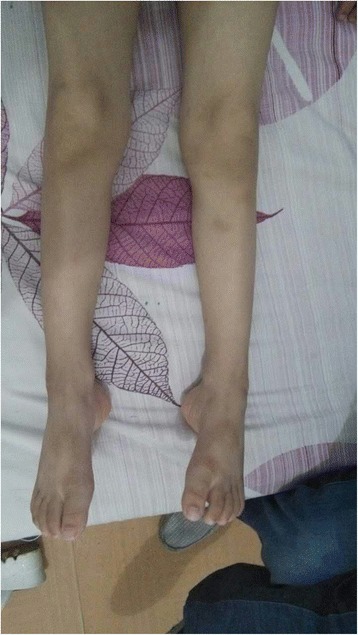
The lower extremities of the patient

The patient was a 4-year-old Chinese boy who couldn’t stand steadily by himself and had suspicious foot-drop gait. Physical examination revealed atrophy of lower extremities (Fig. 1). His left foot showed a mild pescavus deformity and could not lift up by himself. There was no strength in his right hand. Testing of the deep-tendon reflexes showed reduced patellar tendon reflex and ankle reflex. There were no Babinski sign, Chaddock sign, Oppenheim sign and Gordon sign. Muscle strength was reduced with MRC scores of IV on lower extremities. The muscular tension was mildly reduced. NCVS showed damage of peripheral nerves.

**Fig. 2:**
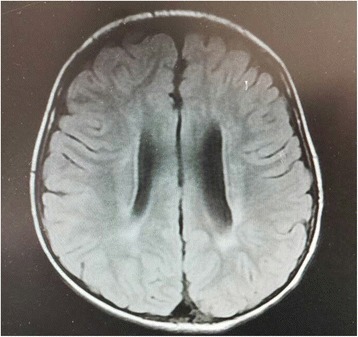
The brain MRI image of the patient

The patient is the third child in his family. His two sisters who are four and two years older were completely healthy. No family history of similar problems was presented. The patient was one of the two twins when his mother was in pregnancy. Unfortunately his twin brother died in the uterus. Since his mother’s amniotic fluid broke early, he was born by caesarean with 5.73 pounds in weight. There was no asphyxia at birth. During his growing progress, he could walk at the age of 1 year and 7 to 8 months albeit not steadily with suspicious foot-drop gait. He was easy to fall when running with progressive aggravation. He had normal intelligence. NCVS showed normal motor nerve conduction velocity (MCV) of upper limbs but showed slower sensory nerve conduction velocity (SCV). In addition, neither compound motor action potential (CMAP) or sensory nerve action potential (SNAP) of lower limbs was elicited, indicating that the peripheral nerves of his lower limbs were damaged (Tables 1 and 2). When it comes to brain and spinal MRI, the brain MRI image indicates the possibility of periventricular leukomalacia (PVL) while the spinal MRI image shows no apparent abnormality (Fig. 2). As for lumbar puncture, the cerebrospinal fluid examination results were normal. The leucocyte count was zero, the protein and sugar quantifications were in normal control range and the immunoglobulin quantifications were as well.

**Table 1:** NCVS results (motor nerve conduction velocity)

Nerve	Stimulation site	Recording site	Latency (mS) & amplitude (mV)				Conduction velocity (m/s)	F wave	
			Proximal muscle		Distal muscle				
			(mS)	(mV)	(mS)	(mV)		mS	Occurrence rate
Right median nerve	Elbow wrist	Abductor pollicis brevis	4	6.3	2	6.7	50		
Right ulnar nerve	Elbow wrist	Abductor digiti quinti	3.7	4.7	1.83	4.7	53.5		
Right tibial nerve	Popliteal fossa ankle	Abductor hallucis muscle	10.8	0.3	Not elicited			Not elicited	
Left tibial nerve	Popliteal fossa ankle	Abductor hallucis muscle	10.7	0.02	5.5	0.02	29.8	Not elicited	
Right common peroneal nerve	Popliteal ankle	Extensor brevis digitorum	Not elicited		Not elicited				
Left common peroneal nerve	Popliteal ankle	Extensor brevis digitorum	Not elicited		Not elicited

**Table 2:** NCVS results (sensory nerve conduction velocity)

Nerve	Stimulation site	Recording site	Latency (mS)	Amplitude (uV)	Conduction velocity (m/s)
Right median nerve	Index finger	Wrist	5.8	5.9	15.5
Right ulnar nerve	Little finger	Wrist	2	42	40
Right nervi suralis	Crus	Ankle	Not elicited		
Left nervi suralis	Crus	Ankle	Not elicited

Since the clinical features of the patient was suspicious of CMT, we sequenced 133 candidate genes for hereditary motor sensory neuropathy (HMSN) and identified a novel heterozygous mutation c.730G > C (p.Val244Leu) in *MFN2*. This mutation was not present in either of his parents.

## Discussion

*MFN2* is located on the short arm of chromosome 1. It encodes mitofusin 2, a GTPase dynamin-like protein of the outer mitochondria membrane. *MFN2* has an essential role in mitochondrial functions including fusion, axonal transport, interorganellar communication and mitophagy [5, 6]. Because mitochondria are crucial organelles present in almost all cells of human body (except erythrocyte) to provide ATP for metabolic processes and oxidative phosphorylation [9], mutations of *MFN2* may cause mitochondria dysfunction and affect high energetic demand tissues. Since the first heterozygous mutation in the *MFN2* has been found by Züchner et al in 2004, more than 100 *MFN2* mutations have been reported to be associated with CMT [6, 7, 10]. Based on the existing reports, these mutations can be either gain or loss of function in mitochondria. In some cases, certain *MFN2* mutations are associated with specific clinical features [11]. c.730G > A (p.Val244Met), a mutation occurring at the same position as the one reported in this study, has been reported to cause CMT2 by K.Kijima et al in 2005. The Japanese kid with the c.730G > A mutation showed CMT2 phenotype with no sensory impairments. The age of onset was 10 months and the symptom included walking difficulties [8]. In our study, the patient’s symptom at the onset was abnormal gait when he started to walk at the age of 1 year and 7 to 8 months. Then he showed running difficulties, frequent falls and slowly progressive atrophy of lower legs with a mild deformity at his left foot. On the other side, this boy did have sensory loss at his lower limbs. All of these symptoms agree with CMT phenotypes. Because of his median nerve conduction velocity is 50.0 m/s (>38 m/s), we classified it as CMT2.

Based on previously reports and the clinical symptoms of the patient, we believe that the novel *MFN2* mutation identified in this study has a deleterious nature and is the likely cause of CMT2 for the following reasons:. First, the c.730G > C mutation changes an amino acid from valine to leucine and may affect protein features. Second, p.Val244Leu in *MFN2* was predicted to be disease-causing by all three computational tools (Mutation Taster, PROVEAN and SIFT). Third, this mutation changed an evolutionarily conserved amino acid (Phylop score is 5.51) (Table 3). Four, according to previous reports, there is one CMT2A disease causing mutation at the same position (c.730G > A/p.Val244Met). In summary, the *de novo* pattern is consistent with the previously reported autosomal dominant inheritance of CMT2A2.

**Table 3:**
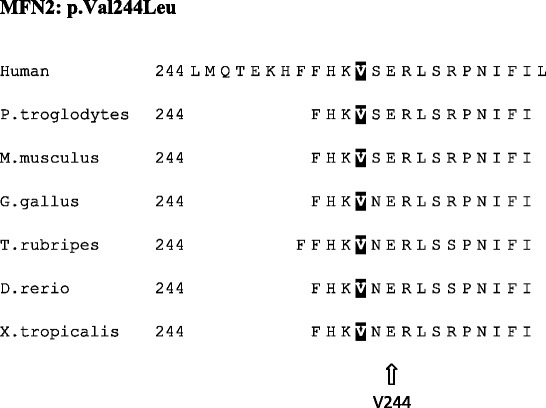
Multi-species sequence alignment showing the evolutionarily conserved residues of p.V244 in *MFN2*

## Conclusion

The c.730G > C (p.Val244Leu) mutation in *MFN2* is a likely pathogenic mutation for CMT2.

### Ethics approval and consent to participate

Written informed consent was obtained from the parents of the patient for participate with ethics approval.

## Consent to publish

Written informed consent was obtained from the parents of the patient for publication of this case report and any accompanying images. A copy of the written consent is available for review by the Editor-in-Chief of this journal.

### Availability of data and materials

The main data that supported the conclusion of the manuscript are presented in the paper and additional supporting files.

## Endnotes

The endnotes we used were reference and included in the reference section.

## Abbreviations

CMT: is the abbreviation for Charcot-Marie-Tooth disease
CMT2: is the abbreviation for Charcot-Marie-Tooth disease type 2
NCVS: is the abbreviation for nerve conduction velocity study
CMAP: is the abbreviation for compound motor action potential
SNAP: is the abbreviation for sensory nerve action potential
HMSN: is the abbreviation for hereditary motor sensory neuropathy
MCV: is the abbreviation for motor nerve conduction velocity
SCV: is the abbreviation for sensory nerve conduction velocity
PVL: is the abbreviation for leukomalacia
